# Biological control agent attack timing and population variability, but not density, best explain target weed density across an environmental gradient

**DOI:** 10.1038/s41598-020-68108-w

**Published:** 2020-07-06

**Authors:** Nathan Harms, James Cronin

**Affiliations:** 1 Aquatic Ecology and Invasive Species Branch, US Army Engineer Research and Development Center, Vicksburg, MS 39180 USA; 2 Department of Biological Sciences, Louisiana State University, Baton Rouge, LA 70803 USA

**Keywords:** Biogeography, Invasive species, Macroecology

## Abstract

Spatial variation in plant–herbivore interactions can be important in pest systems, particularly when insect herbivores are used as biological control agents to manage invasive plants. The geographic ranges of the invasive plant alligatorweed (*Alternanthera philoxeroides*) and its biological control agent the alligatorweed flea beetle (*Agasicles hygrophila*) do not completely overlap in the southeastern USA, producing spatial heterogeneity in interaction strength that may be related to latitude-correlated environmental gradients. We studied this system near the range margin of the alligatorweed flea beetle to test whether spatial variation in alligatorweed density was best explained by agent mean or maximum density, variability in agent density, agent attack timing, or a combination of biological control and environmental (i.e., weather) variables. The pattern that emerged was that mean agent and host densities were negatively and positively associated with latitude, respectively. Variability in agent density increased with latitude and was positively correlated with host density. We further discovered that agent first attack timing was negatively correlated with winter and spring temperatures and spring and summer precipitation, and positively correlated with seasonal temperature extremes, which was then directly influential on agent density and variability in density, and indirectly on host density. This study demonstrates that, contrary to common wisdom, weather-related timing of agent activity and population variability, but not agent mean density, contribute to the spatial heterogeneity observed in alligatorweed populations.

## Introduction

Geographic patterns of species abundance may reflect biotic or abiotic factors that act to delimit their distributions^1,2^. Individuals in populations at the margins of their geographic range may be periodically subject to environmental extremes that meet or exceed their physiological tolerances^3^. Understanding how climate or climate-driven weather events contribute to defining range margins of many organisms, including ectotherms which are especially vulnerable to climatic variation, is important for understanding the ecological and evolutionary implications of climate change^4^. Variation in temperature extremes may lead to shifts in species’ distributions and abundances, generating spatial heterogeneity in the timing and type of species interactions and population dynamics of those species^5–7^.

Interacting species (e.g., mutualists, herbivores and host plants, etc.) may be particularly vulnerable to changing climate if their interactions are modified by increasing mean temperatures or increased climate variability and frequency of extreme weather events^8–10^. One result of climate change may be unequal rates of range expansion/contraction between predators and prey or plants and herbivores, which alters spatial patterns in the timing, frequency and magnitude of their interactions^11–14^. For example, future climate change is predicted to result in a large spatial mismatch between the distributions and abundances of the butterfly *Boloria titania* Esper (Lepidoptera: Nymphalidae) and its host plant *Polygonum bistorta* L. in Europe, but the degree of mismatch may depend on their individual abilities to track changing environmental conditions^13^. Increased variability in biotic or abiotic factors that mediate interactions is expected to lead to more frequent pest outbreaks^15^ and density-dependence among herbivores^16^. This may be especially true in years when regulating effects of abiotic factors (e.g., winter temperatures) are suppressed, leading to higher overwintering survival^16^. A better understanding of variation in herbivore distribution and abundance relative to climate is especially relevant to agronomic or natural systems in which herbivorous pests cause losses^17,18^, or biological control programs in which monophagous herbivores are intentionally introduced to reduce abundance of a pest plant^19^.

Weed biological control has a history of varied successes^20^, some of which can be explained by the different impacts of climate on agent and target weed abundance. For example, both the aquatic weed giant salvinia (*Salvinia molesta*) and its biological control agent, the giant salvinia weevil (*Cyrtobagous salviniae* Calder and Sands; Coleoptera: Curculionidae), are limited by cold temperatures^21–23^. However, the geographic distribution of the weevil is considerably more restricted than its host to lower latitudes, requiring annual reintroduction of the weevil in higher latitudes^23^. Despite its rarity in practice, an explicit examination of these systems from range interior to margin of the agent will provide insight into the relative importance of biotic and abiotic factors on abundance and distribution of agents^24^, and ultimately successful pest control. Predicted patterns of species abundance across their geographic ranges often reflect greater abundance in geographically interior relative to marginal areas but this has received mixed support^25,26^ and may depend on a combination of range size and latitude (i.e., Rapoport's rule)^27–29^. On the other hand, environmental gradients that correlate well with geographic gradients (e.g., the correlation between latitude and temperature) may be a better predictor of species abundance. Low biological control agent abundance might be expected in high-latitude marginal areas because climate variables there are likely to be at or near the agent’s physiological limits. As a result, stochastic events such as extreme weather events should have disproportionate negative effects on biological control agent vital rates in marginal relative to interior areas, leading to reduced pest control.

Here, we examined the role of biotic and abiotic factors on plant–herbivore interactions along an environmental gradient in the USA. This was accomplished through a case study on biological control of alligatorweed (*Alternanthera philoxeroides* (Mart.) Griseb.) by the alligatorweed flea beetle (AFB; *Agasicles hygrophila* Selman and Vogt; Coleoptera: Chrysomelidae) in Louisiana. This system is well-suited to test for patterns of climate variability and related host plant–herbivore densities because the distributions and latitudinal range limits of agent and host are mostly known and it has been observed that host distribution extends much farther north in the USA than the agent. Thus, we would expect abiotic factors to be primarily responsible for shaping the northern distributional limit of the agent. Studying populations near their range margin can be especially valuable for identifying the factors associated with shaping range limits and agent abundance^24^.

We tested the following hypotheses and predictions: (1) mean densities of AFB (Hypothesis 1a) and alligatorweed (Hypothesis 1b) reflect climate-related latitudinal gradients (i.e., AFB decreases with latitude due to increasing climate limitations correlated with latitude; alligatorweed increases with latitude due to decreased control by AFB at higher latitudes); (2) local density of AFB (Hypothesis 2a), but not alligatorweed (Hypothesis 2b), will be more variable in higher relative to lower latitude populations due to occasional temperature exposure at or beyond thermal tolerances of AFB but not alligatorweed; (3) specifically, winter severity is primarily responsible for AFB attack timing on alligatorweed (Hypothesis 3); because of this, (4) winter severity is the best predictor of AFB (Hypothesis 4a) and alligatorweed (Hypothesis 4b) density; and (5) mean AFB density, independent of weather, explains the most variation in alligatorweed density (Hypothesis 5). Although climate-related variability in biological control of weeds has received considerable attention around the world, our study is unique in that we examine direct and indirect relationships between latitude-correlated abiotic factors, an invasive plant, and its biological control agent.

## Results

### Relationship between latitude and agent or host density

**Figure 1:**
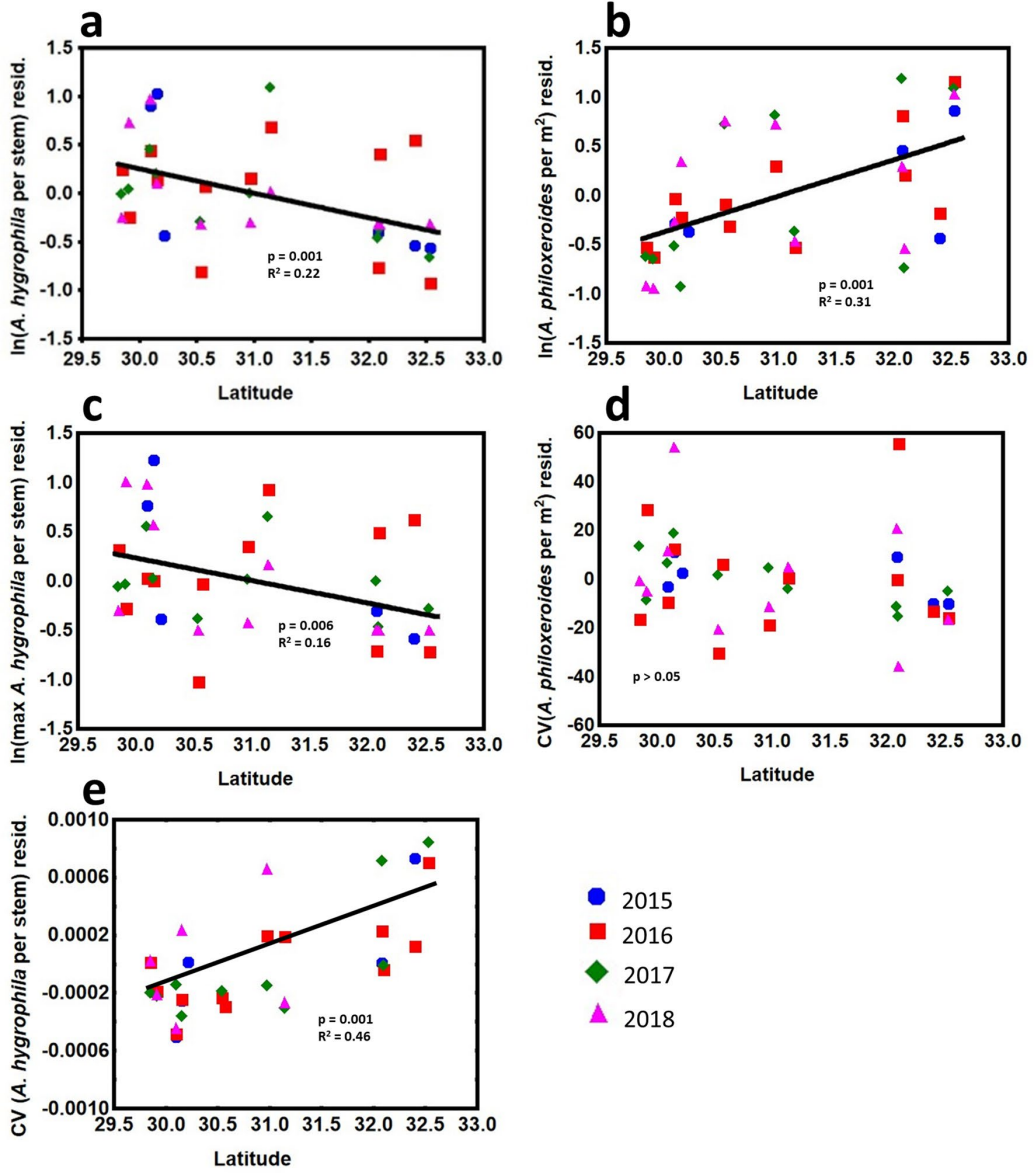
The relationships between latitude and the alligatorweed biological control agent, AFB mean (**a**) and maximum (**c**) density, and variability (**e**), and alligatorweed density (**b**) and variability (**d**) in Louisiana. To emphasize the effect of latitude independent of year, a mixed effects model was conducted without latitude. The residuals from this analysis are plotted against latitude and best-fit lines based on least squares regression are displayed.

Over four years of field measurements to monitor biological control of alligatorweed, latitude was a significant predictor of biological control agent and host abundance. Mean (range − 0.40 ± 0.16 – 0.47 ± 0.15 ln[insects per stem][mean ± s.e.m]) and maximum (range − 0.02 ± 0.24 – 1.57 ± 0.22 ln[insects per stem]) densities of AFB and mean (range: 5.03 ± 0.72 – 5.80 ± 0.27 ln[stems per m^2^]) densities of alligatorweed did not vary significantly among years (Table 1). Additionally, the year × latitude interaction was statistically insignificant. AFB mean and maximum density decreased with latitude (Fig. 1a,c; in support of Hypothesis 1a) and within-year variability (coefficient of variation) of AFB mean density increased with latitude (Fig. 1e; in support of Hypothesis 2a). Alligatorweed density increased with latitude (Fig. 1b; in support of Hypothesis 1b), but variability did not (Fig. 1d; in support of Hypothesis 2b). Overall, plant and insect densities were correlated (alligatorweed mean density – AFB mean density, *r* = − 0.51; alligatorweed mean density – AFB maximum density, *r* = − 0.45).

**Table 1:** Results for mixed effects models to examine the importance of latitude and year on alligatorweed and AFB density and variability across Louisiana.

	Alligatorweed density			Alligatorweed CV			Maximum AFB density			Mean AFB density			AFB CV		
Effect	df	F	*P*	df	F	*P*	df	F	*P*	df	F	*P*	df	F	*P*
Latitude	1,30	13.53	** < 0.001**	1,30	2.00	0.17	1,30	8.60	**0.006**	1,30	10.54	**0.002**	1,30	13.75	**0.001**
Year	3,30	0.26	0.85	3,30	0.53	0.67	3,30	1.66	0.20	3,30	0.71	0.56	3,30	0.27	0.84
Latitude × Year	3,30	0.22	0.88	3,30	0.56	0.65	3,30	1.90	0.15	3,30	0.78	0.51	3,30	0.27	0.85

n = 38, CV = Coefficient of variation.

### Biotic and abiotic predictors of alligatorweed density

**Figure 2:**
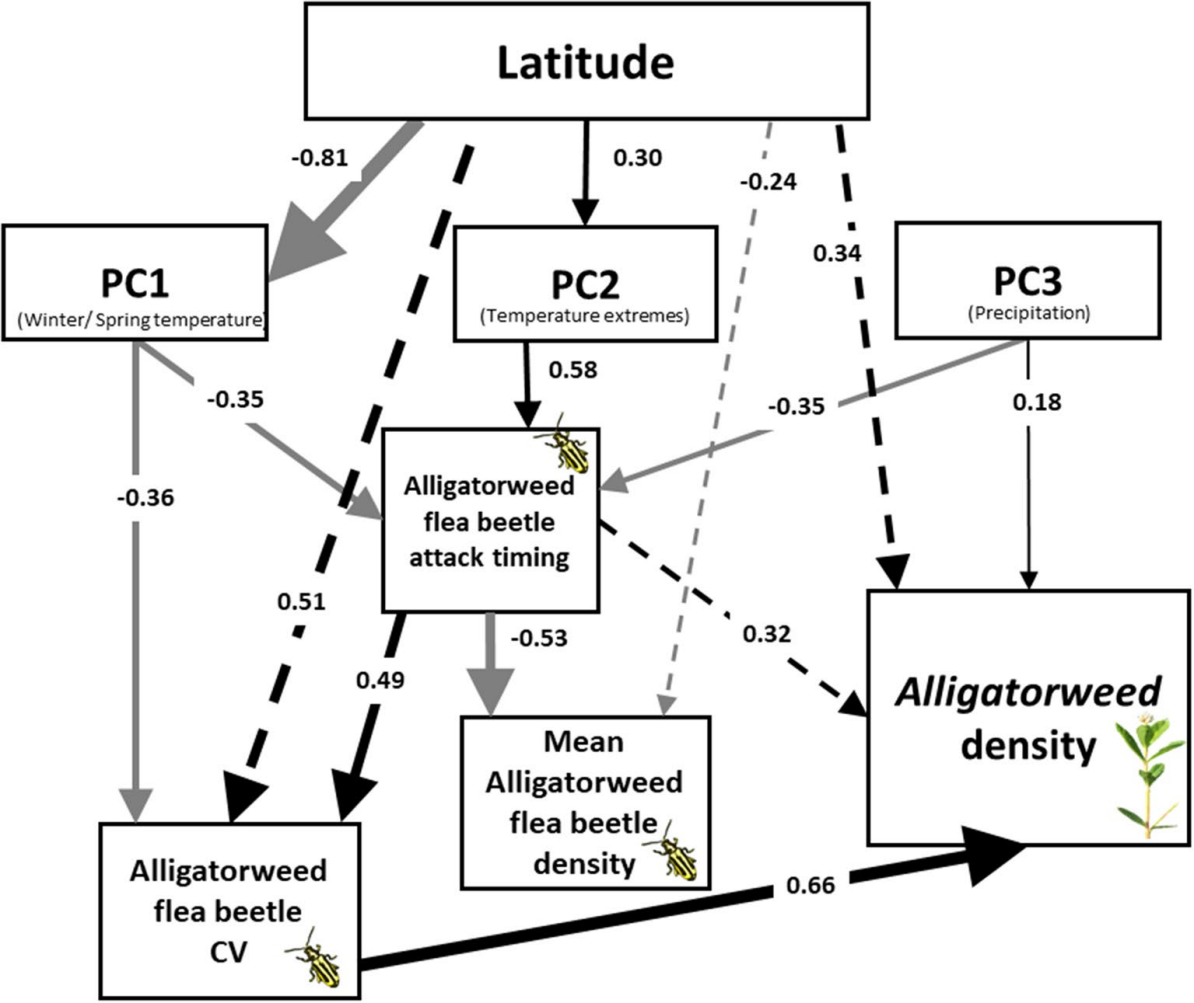
Best-fit model 1 (Table 2) to explain alligatorweed density in relation to biological control agent abundance, variability and timing, and weather. Solid lines are significant direct effects and dashed lines are indirect effects between variables. Standardized path coefficients are given for each path. For clarity, positive paths are shown in black and negative paths are shown in gray.

Alligatorweed density in the northern range of its primary biological control agent was explained by three equally likely models based on AICc (Table 2; Appendix 3). Based on a combination of AICc and other fit metrics, we selected a top model (Fig. 2) that largely demonstrated the direct effects of weather on biological control agent but not plants (Appendix 3). The top model explained a substantial part of the total variance for AFB mean density (R^2^ = 0.28) and variability (R^2^ = 0.49), the date of first biological control activity (R^2^ = 0.59), and alligatorweed mean density (R^2^ = 0.43). In contrast to the bivariate analysis above, latitude did not have a direct effect on either agent or plant density, but was influential through indirect effects on both agent and plant density as mediated by weather variables. Weather variables had little to no direct effect on alligatorweed density over the study region and only the direct path between PC3 and plant density was retained in the final model. Specifically, winter severity was not directly important for alligatorweed (rejection of Hypothesis 4b) or AFB (rejection of Hypothesis 4a) densities, except indirectly through mediation of attack timing (see below). In contrast, alligatorweed density had a strong positive relationship to variability in AFB density and attack timing, but not AFB mean density (rejection of Hypothesis 5) so this path was removed from the final model. Latitude was indirectly related to AFB density and variability, and alligatorweed density through its relationship to weather variables.

**Table 2:** Model rank and fit indices for a subset (best and full models) of model combinations.

Model Rank	*Ah* Mean/Max	AICc	ΔAICc	Likelihood	Akaike Wt	RMSEA	GFI	AGFI	χ ^2^
1	Mean	65.93	0.00	1.00	0.52	0.085	0.88	0.73	0.23
2	Max	67.18	1.25	0.53	0.28	0.10	0.87	0.72	0.15
3	Mean	67.85	1.92	0.38	0.20	0.079	0.89	0.73	0.26

The top model is highlighted and was chosen based on a combination of model selection (AICc), absolute fit (χ^2^) and relative-fit (GFI, AGFI) indices. The first column denotes whether the model had a mean (Mean) or maximum (Max) density variable for *A. hygrophila* (*Ah*). AICc = Akaike Information Criterion corrected for small samples size, ΔAICc = difference between AICc of the model and AICc of the top model, RMSEA = root mean square error approximation, GFI = Goodness-of-fit index, AFGI = Sample-size adjusted goodness-of-fit index, χ^2^ *P* = Chi-square probability.

Latitude-correlated weather variables are clearly important to AFB performance in marginal areas. All three weather PCs were influential for timing of AFB activity (Fig. 2). In particular, winter and summer severity (PC2) had the strongest effect on activity timing, which supports our hypothesis about the importance of winter on subsequent activity and population growth of AFB (Hypothesis 3). Mean density of AFB was negatively related to attack timing and latitude. Variability of AFB density was negatively related to winter and spring temperatures (PC1) and positively related to timing of activity. In agreement with the bivariate analysis, variability of AFB density was also positively related to latitude (Hypothesis 2a). Contrary to our prediction, foliar nitrogen was not an important variable in the analysis and was not retained in the top models.

## Discussion

We found that the density of an invasive aquatic weed, alligatorweed, was largely determined by biotic factors such as phenology (attack timing) and annual variability of its biological control agent but not agent density. In contrast, agent density was determined primarily by temperature through the influence on the timing of agent herbivory. For tightly-interacting species, such as specialized biological control agents and their host plants, the relative importance of factors that shape their abundance patterns and range margins may be spatially heterogeneous^13,30^. Studying organisms across their geographic range, with an explicit inclusion of marginal areas, allows for the identification and measurement of these factors and provides an opportunity to make predictions about future interactions with climate change^24^.

Altered timing of herbivore-plant interactions is a critical prediction of the ecological effects of climate change^4,7,14,31–34^. Warmer winter temperatures at high latitudes may lead to range expansion mediated by increased winter survival in herbivores and their hosts. In cases where there is already some degree of phenological mismatch between herbivore and host, small changes in the timing of the interaction could be dire for herbivore populations^35^. For example, a pair of lepidopteran species were shown to frequently suffer high mortality because of mismatches in the seasonal timing of their early egg hatch (*Operophtera brumata* L.; Lepidoptera: Geometridae) or late eclosion (*Euphydras editha bayensis* Boisduval; Lepidoptera: Nymphalidae) with the presence of the suitable life stage of their host plants (*Quercus robur* for *O. brumata*, *Plantago* or *Castilleja* for *E. editha*). Further increases in asynchrony between lepidopteran and plant life histories (e.g., as a result of climate change) will likely lead to more frequent population extinctions of the herbivore^35^. On the other hand, warming temperatures may not disrupt plant–herbivore relationships, but only advance their timing within the year^31,36^. Phenology of *Corythucha ciliata* (Say) (Hemiptera: Tingidae) and its host plant *Platanus x acerifolia* (Platanaceae) (London plane) both responded similarly to experimental warming, with an advance of post-overwintering activities for *C. ciliata* and leaf expansion for *P.* x *acerifolia* in spring during the study. Although phenological synchrony between herbivores and hosts may be maintained for the near future, a plastic response (i.e., earlier activity) by *C. ciliata* to warming is thought to increase the likelihood of future outbreaks by increasing insect population size early in the year^31,37^. Whether or not individual herbivore-host systems will be drastically altered may be related to the relative importance of climate variables on each of the interacting species, which itself depends on location within the ranges of the species^5^.

It is now well understood that factors responsible for limiting populations at high latitudes may not be as important as at low latitudes^2,38^. For instance, the stress-trade-off hypothesis suggests a general rule that abiotic stressors such as temperature limit species distributions in harsh environments (i.e., high latitudes) and that biotic interactions have a larger influence in benign environments (i.e., low latitudes)^2,39–41^. Although evidence in support of this hypothesis is mixed^42^, it is clear that different variables can influence species density in different parts of the geographic range. For example, in the forest pest *Dendroctonus frontalis* Zimmerman (Coleoptera: Scolytinae) (the southern pine beetle), winter severity explained a sizeable portion of the variation associated with population dynamics in northern but not southern locations^5^. In the same study, it was demonstrated that supercooling point (a metric of cold-hardiness) of a northern *D. frontalis* population was significantly lower than a southern population, apparently the result of an adaptive response to more consistently low temperatures in high latitudes. Our study was conducted across a large portion of the latitudinal range of AFB in the USA, and included its northern range limit, but we did not survey a large enough area to determine which factors were responsible for the southern range limits of AFB (*or* northern range limits of alligatorweed*)*. However, our findings suggest that the system is inherently climate-limited in that variables such as winter and spring temperatures, and winter and summer temperature extremes, influenced the timing, variability, and density of AFB, which in turn influenced alligatorweed density.

With global climate change, there is an expected increase in mean temperatures and variability of seasonal conditions (e.g., more frequent extreme events), with complex impacts on performance of organisms^43–46^. Given that we found temperature (PCs 1, 2) to be important for predicting the timing of flea beetle activity, warming climate should lead to earlier timing of flea beetle activity in high latitudes and associated decreased abundance of alligatorweed. However, if climate variability increases, as is predicted^47^, then negative impacts of increasingly-frequent extreme weather events (i.e., climate variation) may outweigh the benefits of consistently warmer mean winter and spring temperatures^44,48^. Additionally, the relative importance of climate variability on performance of a species may reflect the latitudinal origin of that species^49^. Across a number of taxonomic groups, higher latitude species typically have broader thermal tolerances than low-latitude species^50^, but this range in tolerances is almost always skewed towards low temperatures such that upper thermal limits do not appreciably change with latitude but lower thermal limits do change. Because AFB is a tropical/ subtropical species, it may not possess the thermal adaptations to survive increased climatic variation in areas at the range margin ^49–52^. Therefore, additional releases in the USA of AFB from different parts of the native range may provide the genetic variation needed to promote adaptation to high latitude climates. Additional research is needed to better predict how differences in temperature means and variability will impact future biological control of alligatorweed given current or future biological control agents.

In both bivariate and multivariate analyses, we demonstrated a latitudinal pattern in agent and plant density that was consistent with theory and other observations of density patterns across environmental gradients^53–55^. That alligatorweed density increased with latitude suggests that the benefit of reduced biological control at high latitudes outweighs potential limitations from weather. This study also highlights the value of biological control and the importance of agent phenology for explaining abundance of the host plant. In future climates and with associated variability in weather events, systems like this one may experience increasingly variable control efficacy due to changes in timing of agent activity and abundance. In these programs, consideration of new agents sourced from climatically-similar areas of the native range may be warranted. Additionally, the results of this study may assist other programs in which variable control is observed, especially where agent and host geographic distributions are not fully-overlapping and limiting environmental gradients are suspected.

## Methods

### Study system

Alligatorweed is a South American aquatic clonal plant, introduced into the USA during the twentieth century and currently present in the southeastern USA^56^ and California^57^. In the 1960s, AFB, alligatorweed thrips (*Amynothrips andersonii* O’Neill; Thysanoptera: Phlaeothripidae), and alligatorweed moth (*Arcola* (= *Vogtia*) *malloi* Pastrana; Lepidoptera: Crambidae) were released in the USA^56^. Source populations for the original introductions of AFB in the USA were from Ezeiza Lagoon, near Buenos Aires, Argentina (~ 34.5°S; released in California and South Carolina in 1964) and areas near Montevideo, Uruguay (~ 35°S; released in South Carolina in 1964)^58^. Although alligatorweed and its biological control agents are present throughout the southeastern USA, the plant has a broader distribution than its control agents^59,60^. In particular, alligatorweed can be found in the USA at least as far north as Kentucky (36.9°N)^61^, but overwintering of AFB is limited to areas that remain warm during winter (variously reported as mean minimum winter temperatures > 8.9 °C^58^, > 10 °C^59^, or > 11.1 °C^62^ in the USA and > 6.7 °C in China^63^), which roughly corresponds to 32°N in Louisiana, USA.

### Alligatorweed and AFB density along a latitudinal transect in Louisiana

**Figure 3:**
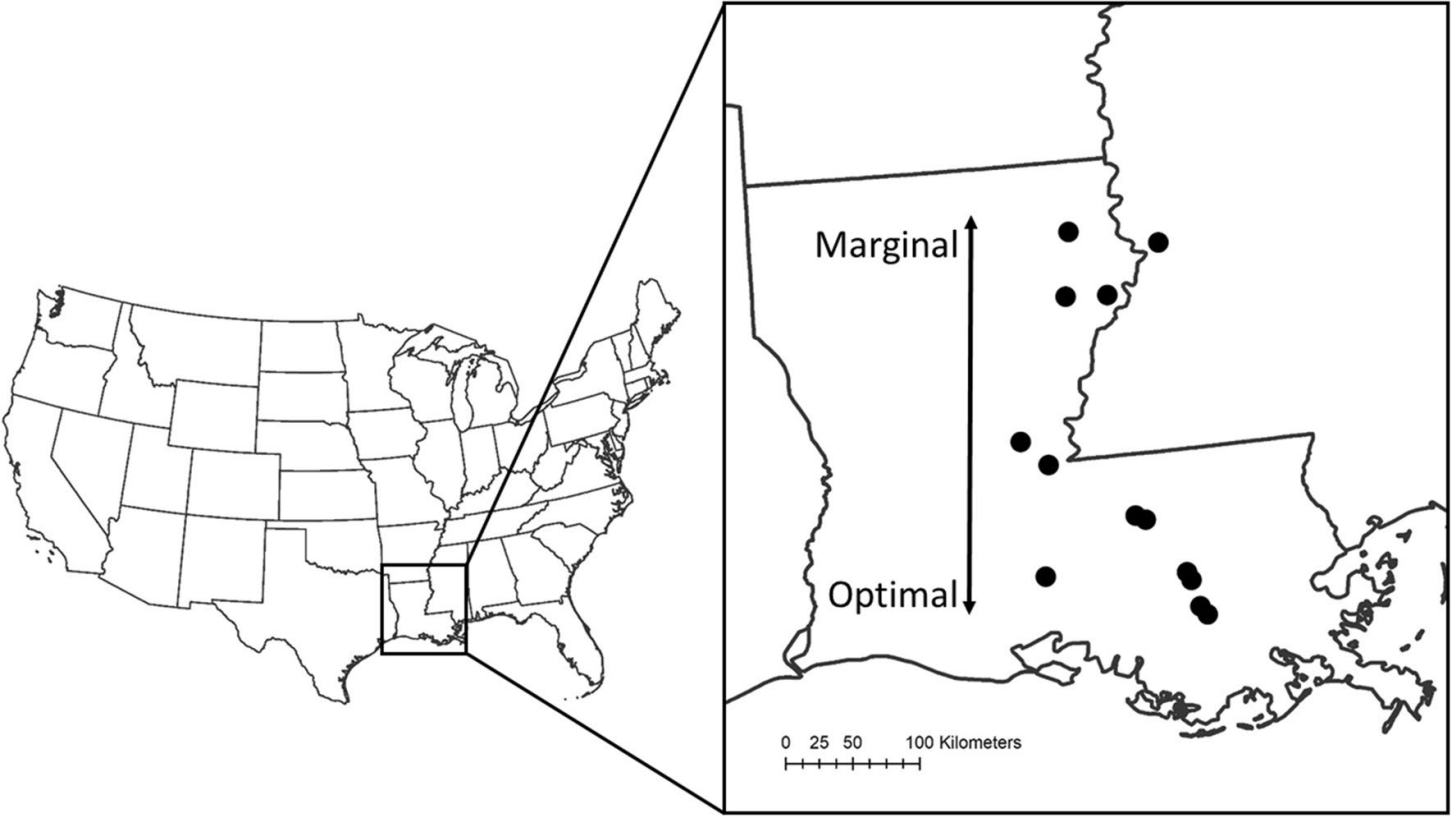
Locations in Louisiana, USA where biological control of alligatorweed was monitored over four years. Marginal and optimal habitats for the biological control agent AFB are labeled.

From 2015 to 2018, we monitored biological control of alligatorweed along a latitudinal transect (29.8–32.5°N) in Louisiana, which encompasses most of the latitudinal range of AFB in North America (Fig. 3). In 2015, we surveyed six sites and in 2016–2018 we surveyed 9–12 sites spanning the range of AFB in Louisiana (Appendix 1). Locations were visited every 2–3 weeks beginning in February or March of each year through October or November, for a total of 353 site visits over four years. During 2015, visits ended in September because of site access issues due to flooding. During visits, plant density was estimated by placing a 1/10 m^2^ (32 cm L × 32 cm W) PVC quadrat in four locations within alligatorweed infestations then counting all emergent alligatorweed stems. Mean plant density (number of alligatorweed stems per 1/10 m^2^) and variability in plant density (coefficient of variation) was calculated for each site and year. Because timing of growth and abundance of plants varied among sites based on latitude and conditions each year, we used a single mean density estimate for annual alligatorweed abundance in each site. Additionally, leaves were collected on most sampling dates for foliar nitrogen determination as described previously^64^, in which foliar nitrogen was important for AFB development and survival.

For each visit, AFB density was estimated. Ten to twenty alligatorweed stems (Mean ± SE: 17.8 ± 0.2 stems) were collected, placed in plastic zip top bags, and examined within 24 h. AFB larvae, pupae, and adults were counted for each stem. When entrance or exit holes were observed, stems were dissected to detect larvae, pupae, and adults. Total density of AFB is reported based on the sum of all life stages per stem. In addition to mean herbivore abundance during the year, rapid defoliation events caused by biological control agent outbreak events may be important for successful biological control^65^. Therefore, in addition to mean density we used maximum biological control agent density in statistical models. Mean density for both agent and host was calculated first as the averaged sum of individuals per stem (or stems per m^2^) on a particular sampling date and site, then averaged for each site and year. Maximum density of AFB was determined from mean abundances for each site and sampling dates. Within-year variability in agent or host density was calculated for each site and year as the coefficient of variation from site visit data within that year.

### Weather data

Although winter temperatures are thought to be the primary determinant of AFB density, other seasonal factors may contribute to AFB population dynamics across its range, particularly maximum summer temperatures and humidity^66^. Therefore, we obtained additional weather data (winter minimum temperature, spring minimum/maximum/average temperature, spring precipitation, summer maximum temperature, summer precipitation) from 82 weather stations within the state of Louisiana for 2014–2019 from the National Oceanic Administration Agency (NOAA) National Centers for Environmental Information (NCEI) online climate database (ncdc.noaa.gov). Relevant weather variables were selected based on previous experience in this system or literature review. In particular, winter temperatures may limit overwintering of AFB, spring weather may explain beetle activity and control of its host^58,62^, and summer maximum temperatures likely limit activity of the agent through impacts on egg hatching and fecundity^67^. Average daily minimum temperatures were calculated from November 1 to March 1 each year. A winter severity index (WSI) was also calculated, equal to the number of days with minimum temperatures below freezing. Average spring temperatures were calculated as the daily minimum/maximum average, then averaged over the period March 1 until June 1. Maximum daily summer temperatures were the average maximum daily temperature between June 1 and September 1 each year. Weather data were calculated for each weather station then we spatially interpolated study site-specific weather information by kriging in ArcMap v10.5 (ESRI, Redlands, California)^68^.

**Figure 4:**
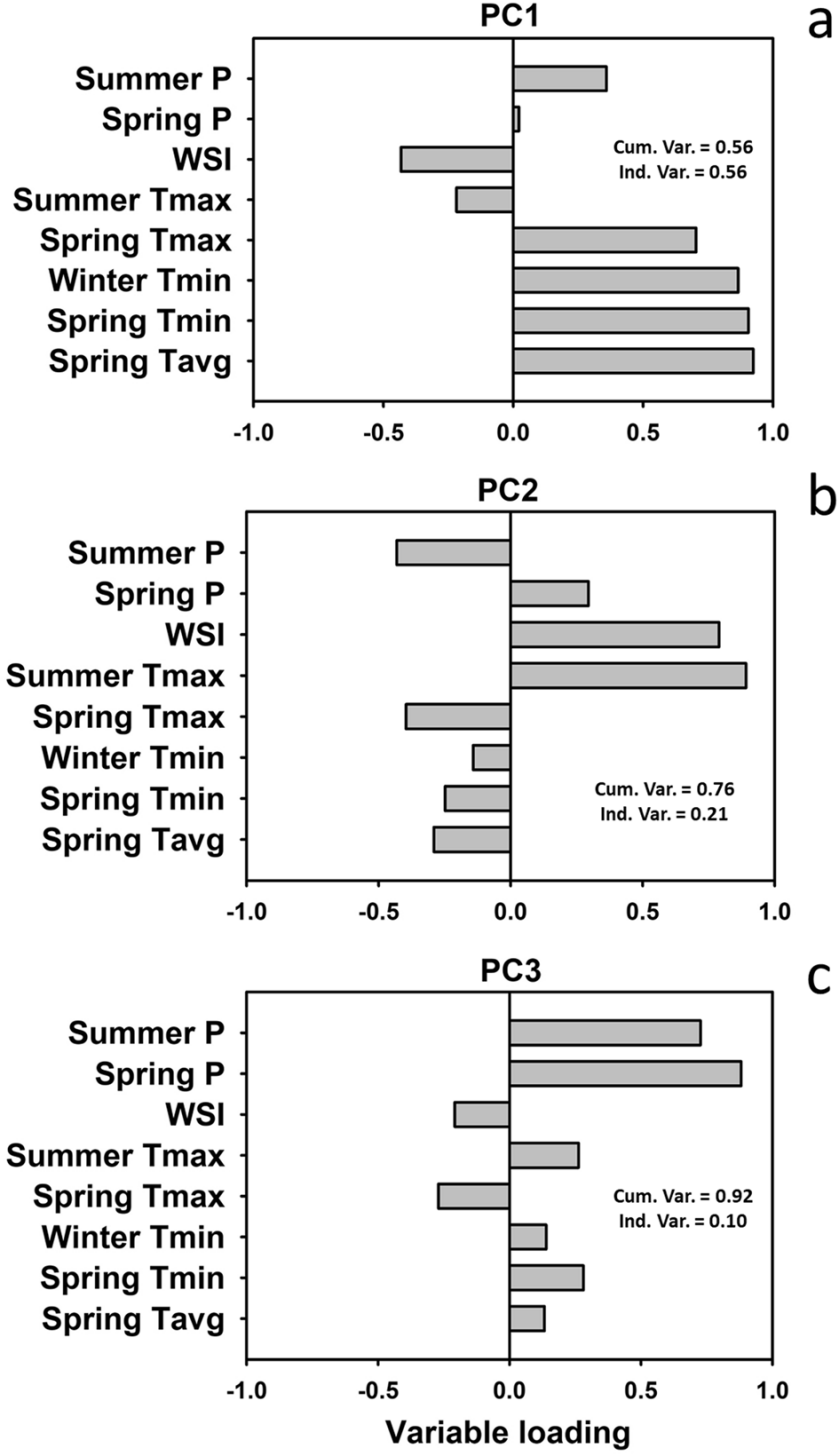
Results of principal components analysis and weather variable loadings for each of three principal components. Cumulative and individual variance explained by each PC is included in each panel. PC1 (**a**) was positively correlated with winter and spring temperatures, PC2 (**b**) was positively correlated with extreme seasonal temperatures (i.e., in winter and summer), and PC3 (**c**) was positively correlated with precipitation in spring and summer. P = Precipitation, WSI = winter severity index, T_max_ = maximum daily temperature, T_min_ = minimum daily temperature, T_avg_ = average daily temperature.

The eight weather variables were standardized, then reduced to three principal components (PC1-3; Fig. 4) using PROC FACTOR in SAS version 9.4 (SAS Institute, Cary, North Caroline). The retention of three variables was based on examination of eigenvalues and the scree diagram^69^. The three PCs represent independent linear combinations of the weather variables and accounted for 92% of total variance present in the original variables. Although PC3 had an eigenvalue less than one (0.79), it was retained because of the high loadings associated with precipitation not present in PC1-2. PC1 was positively correlated with winter and spring temperatures, PC2 was positively correlated with winter severity and summer maximum temperature (i.e., extreme temperatures), and PC3 was positively correlated with spring and summer precipitation (i.e., precipitation) (Fig. 4). All three PCs were used in structural equation modeling described below.

### Statistical analyses

#### Relationship between latitude and agent or host density

To determine whether alligatorweed biological control varied along a latitudinal transect, we used separate mixed effect models. In these models, AFB mean or maximum density and alligatorweed mean density were the dependent variables, latitude was a continuous predictor, year was a main effect, and the latitude × year interaction was included. Distributions of density variables were normalized through natural log (+ 0.5) transformation before analysis. Transformation of coefficient of variation values was not necessary.

#### Direct and indirect effects of latitude and weather on AFB and alligatorweed density

We further explored direct and indirect effects of latitude and weather on agent or host density using structural equation modeling (SEM). Structural equation modeling, also known as ‘modern path analysis’, is a statistical approach to determine the direction and magnitude of relationships (including direct and indirect effects) between multiple associated variables, equivalent to a series of linear models^70^. We used SEM to assess the importance of weather on biological control agent phenology (i.e., attack timing) and density and the dual importance of weather and biological control on host plant density. Based on our knowledge of the alligatorweed biological control system, we first generated a path diagram to depict the full conceptual model, including all measured or estimated weather, insect, and plant variables with direct and indirect interactions (Appendix 2). In the full model, covariance parameters were added between weather-related PCs and between AFB density and variability. Because maximum and not mean herbivore density may be more important to alligatorweed population density, we also constructed a second set of models using the same SEM approach but replaced mean AFB density with maximum AFB density, retaining all other connections and variables. The full model hypothesizes all PCs are correlated with latitude, and they directly influence AFB and alligatorweed densities. Specifically, PC1 and PC2 are likely to influence the timing of AFB activity because winter and spring (and extreme) temperatures have been previously reported as important^71,72^. PC3 may be important if spring and summer precipitation leads to increased humidity or has a positive influence on plant quality, which is critical for larval survival and development^73^. AFB density should have a direct effect on alligatorweed density. Additionally, timing of AFB activity should have a strong indirect effect on alligatorweed because it has been suggested that timing of AFB attack, rather than absolute density was critical for control^74^. Foliar nitrogen was predicted to positively relate to AFB density based on previous work^64^. Prior to SEM analysis, all variables except PCs were standardized to Z-scores^75^; PCs were generated based on already-standardized variables.

To determine the model that best explained alligatorweed density from a subset of models that included the full models (one with mean AFB density and one with maximum AFB density), we used an iterative approach coupled with absolute and relative best-fit indices^76^. Data were fit using the maximum likelihood estimation method. From the full models, we examined results of Wald Chi-squared tests to determine which relationships did not contribute to the model^77^. Parameters with statistically insignificant univariate probabilities (i.e., *P* ≥ 0.05) were removed from the model. We removed a single parameter at a time, reassessing parameter significance each time. From the models generated by variable removal (a total of 34 model iterations), we used Akaike Information Criterion adjusted for small sample size (AICc)^78^ to select the most informative models from the set of full and partial model combinations^76^. ΔAICc was calculated as the difference between the top model and all others. Models with ΔAICc ≤ 2 were considered to have substantial support^78^. Akaike weights are also reported, which represent the relative likelihood that the model is the best given the data and other candidate models. Next, absolute model fit was assessed for the full model and models with ΔAICc ≤ 2 from the top model. This was done using Chi-square lack-of-fit *P*-values, goodness-of-fit index adjusted for degrees of freedom (AGFI), and root mean square error of approximation (RMSEA)^76^. Good model fit is indicated by Chi-square lack-of-fit *P*-values > 0.05, AGFI ≥ 0.9, and RMSEA ≤ 0.08. SEM analysis and model fit parameters were determined in SAS using PROC CALIS.

## Supplementary information

Supplementary information

## Data Availability

Data are available upon request to the first author.
