# Supplementary material for: Biological control agent attack timing and population variability, but not density, best explain target weed density across an environmental gradient
Source: Sci Rep. 2020 Jul 6;10:11062. doi: 10.1038/s41598-020-68108-w (PMC7338522; doi:10.1038/s41598-020-68108-w)
Supplement: Supplementary file 1 — Supplementary information [file 41598_2020_68108_MOESM1_ESM.pdf]

Supplemental Information for:

Biological control agent attack timing and population variability, but not density, best explain target weed density across an environmental gradient

Nathan Harms[1,2,*], James Cronin[2]

[1]US Army Engineer Research and Development Center, Vicksburg, MS 39180 USA
[2]Louisiana State University, Department of Biological Sciences, Baton Rouge, LA
*Corresponding author: Nathan.E.Harms@usace.army.mil

Appendix 1.

Table A1. Locations where alligatorweed biological control was monitored for this study.

Location	Latitude	Longitude	Waterbody type	Years sampled
Choctaw Boat Ramp, LA	29.850	-90.679	River	2016, 2017, 2018
Bayou Chevruil, LA	29.912	-90.729	River	2016, 2017, 2018
Blind River, LA	30.095	-90.779	River	2015, 2016, 2017, 2018
Marepaus Wildlife Management Area, LA	30.150	-90.807	Swamp	2015, 2016, 2017, 2018
Martin Lake, LA	30.215	-91.900	Lake	2015
Blackwater Conservation Area, LA	30.535	-91.089	Wetland	2016, 2017, 2018
Greenwood Community Park, LA	30.570	-91.167	Pond	2016
Simmesport Pond, LA	30.969	-91.808	Pond	2016, 2017, 2018
Spring Bayou, LA	31.142	-92.009	River	2016, 2017, 2018
Lake Saint Joseph, LA	32.077	-91.233	Lake	2015, 2016, 2017, 2018
Bayou Macon, LA	32.094	-91.564	River	2016, 2017, 2018
Openwood Pond, MS	32.396	-90.794	Pond	2015, 2016
Poverty Point Reservoir, LA	32.529	-91.495	Reservoir / Lake	2015, 2016, 2017, 2018

Appendix 2.

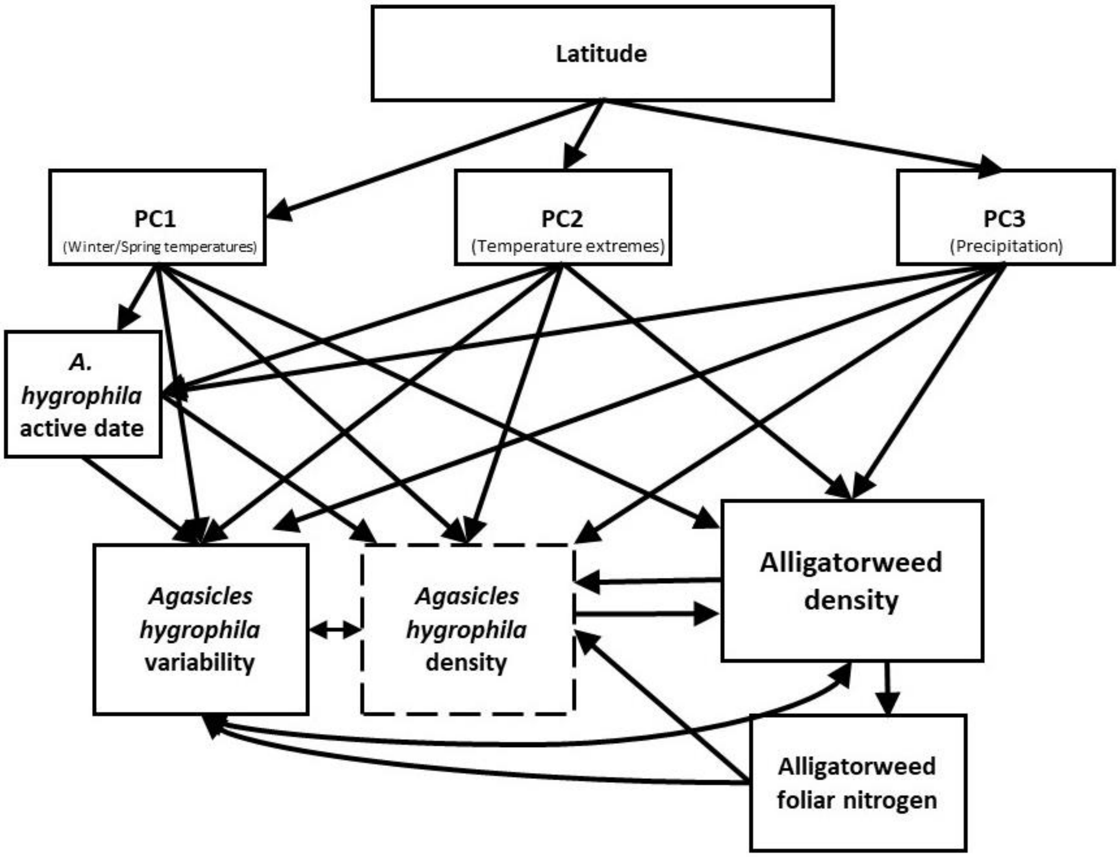

Figure A2.1. Conceptual model of factors determining *A. philoxeroides* abundance in a biological control system. The dashed box around the *A. hygrophila* density variable is to denote that two separate conceptual models were considered- one in which mean density was the *A. hygrophila* density variable and one in which maximum density was the *A. hygrophila* density variable. The conceptual model was used as the full model for subsequent selection and examination of parameter significance. Indirect effects are not shown in this diagram.

Appendix 3.

Table A3.1. Model fit and comparison results for *A. philoxeroides* and biological control. Model 1 is presented in the text, full models are presented in Appendix 2 and models 2-3 are below. N = number of observations, K = number of estimated parameters, AIC = Akaike Information Criterion, AICc = AIC corrected for small sample size, ΔAICc = difference between AICc of the model and AICc of the top model, RMSEA = Root Mean Square Error Approximation, GFI = Goodness-of-fit index, AGFI = sample size corrected GFI, χ^2 *P* = Chi-square probability.

**Model**	**N**	**K**	**AIC**	**ΔAIC**	**AICc**	**ΔAICc**	**Likelihood**	**Akaike Wt.**	**RMSEA**	**GFI**	**AGFI**	χ^2
1-Mean	39	9	59.72	0.00	65.93	0.00	1.00	0.52	0.0853	0.8818	0.7341	0.233
2-Max	39	9	60.97	1.25	67.18	1.25	0.53	0.28	0.1048	0.8671	0.7187	0.1501
3-Mean	39	10	59.99	0.27	67.85	1.92	0.38	0.20	0.0789	0.8893	0.7343	0.2632
Full-Max	39	23	89.35	29.63	162.95	97.02	0.00	0.00	0.1702	0.9053	0.5265	0.0435
Full-Mean	39	23	89.66	29.94	163.26	97.33	0.00	0.00	0.17	0.9039	0.5193	0.0394

Table A3.2 Parameter estimates and significance terms (*P*-values) for direct and indirect effects in the selected best model. PC1-3 are principal components of weather variables, *Ah* = *A. hygrophila*, *Ap* = *A. philoxeroides*, CV = Coefficient of variation.

	Path			Estimate	*P*
**Direct effects**	Latitude	===>	PC1	-0.81	<.0001
	Latitude	===>	PC2	0.3	0.06
	PC1	===>	Attack timing	-0.35	0.002
	PC1	===>	CV *Ah* density	-0.36	0.003
	PC2	===>	Attack timing	0.58	<.0001
	PC3	===>	Attack timing	-0.35	0.002
	CV *Ah* density	===>	Mean *Ap* density	0.66	<.0001
	Attack timing	===>	Mean *Ah* density	-0.53	<.0001
	Attack timing	===>	CV *Ah* density	0.49	<.0001
**Indirect effects**	Latitude	===>	Mean *Ap* density	0.34	<0.001
	Latitude	===>	CV *Ah* density	0.51	<0.001
	Latitude	===>	Mean *Ah* density	-0.24	0.01
	Attack timing	===>	Mean *Ap* density	0.32	0.001

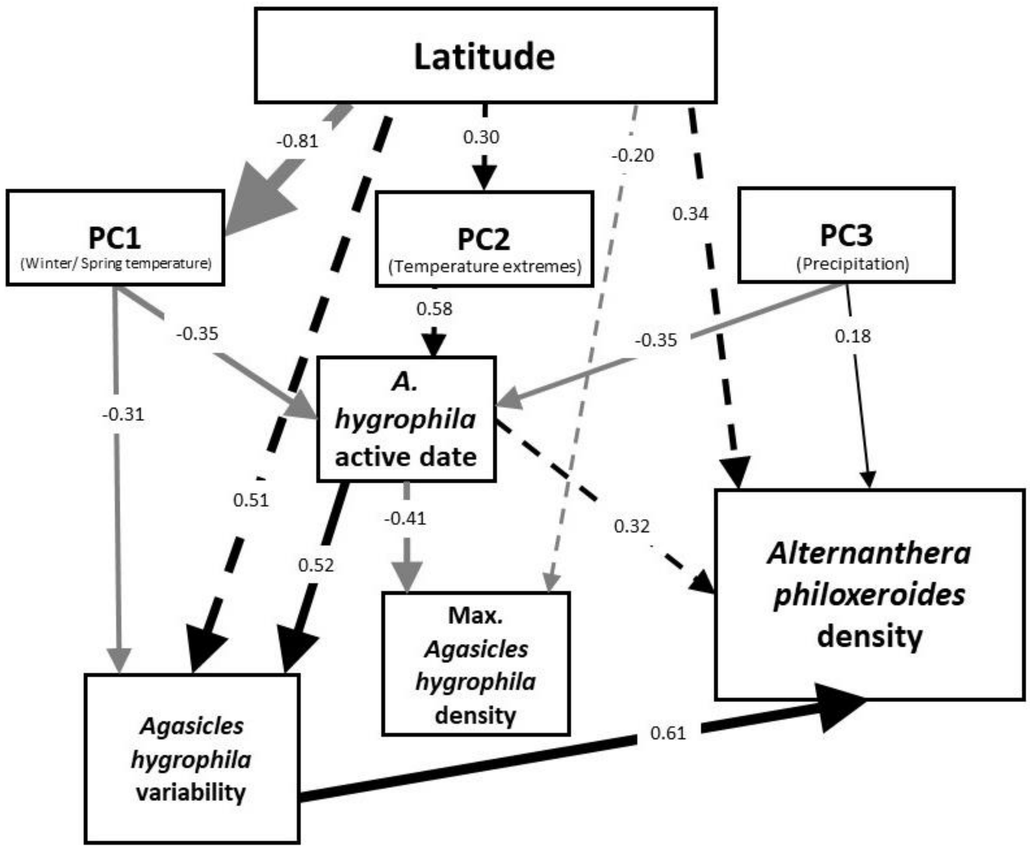

Figure A3.2. Model 2 with maximum *A. hygrophila* density as the biological control agent abundance variable.

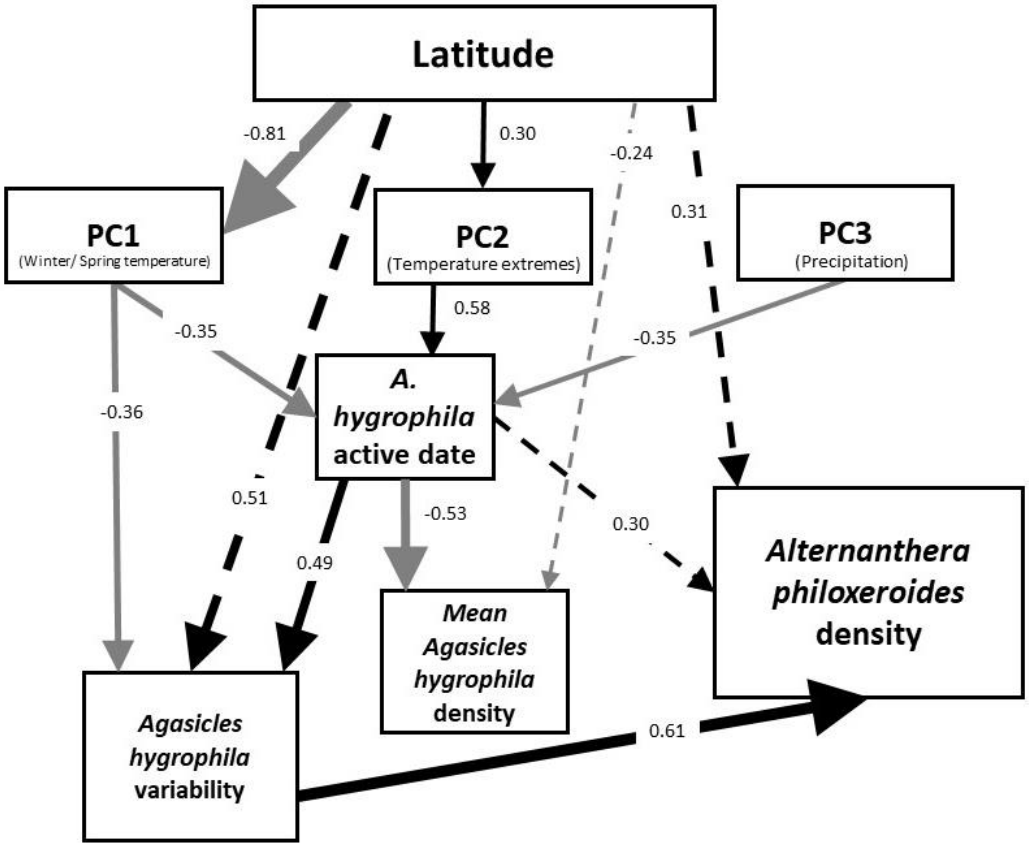

Figure A3.2. Model 3 with mean *A. hygrophila* density as the biological control agent abundance variable.
